# Influence of sex on renin‐angiotensin‐aldosterone system metabolites and enzymes in Doberman Pinschers

**DOI:** 10.1111/jvim.16589

**Published:** 2022-11-22

**Authors:** Darcy B. Adin, Jorge A. Hernandez

**Affiliations:** ^1^ Department of Small Animal Clinical Sciences University of Florida, College of Veterinary Medicine Gainesville Florida USA

**Keywords:** ACE, Doberman Pinscher, estrogen, genotype, RAAS

## Abstract

**Background:**

Estrogen modulates the renin‐angiotensin‐aldosterone system (RAAS) in women, but sex differences have not been fully explored in dogs.

**Objective:**

We hypothesized that the RAAS profile of intact female (IF) Doberman Pinschers (DP) would differ from spayed female (SF) and intact male (IM) DP.

**Animals:**

Eighteen healthy DP (6 IF, 6 SF, 6 IM).

**Methods:**

Absolute and indexed RAAS metabolites, angiotensin‐converting enzyme (ACE) and ACE2 activities, and genotypes (pyruvate kinase dehydrogenase 4, titin, and ACE variants) were compared among sex groups using Kruskal‐Wallis or chi‐square tests, and linear regression controlling for age. Data are expressed as median (minimum, maximum) and *P* < .05 was considered significant.

**Results:**

The ACE activity was higher in IF DP (656 pmol/L; 436, 784) compared to SF DP (411 pmol/L; 287, 451; *P* = .01) and IM DP (365 pmol/L; 276, 1200; *P* = .04) after controlling for age. Angiotensin II, angiotensin I, and plasma renin activity marker (PRA‐S) were higher in IF DP compared to SF DP, but not significantly (*P* ≤ .25). After controlling for age, angiotensin 1‐7/angiotensin I was lower in IF DP compared to SF DP (*P* = .01). Genotypes did not differ among groups. Most DP (94%) were ACE variant positive.

**Conclusions and Clinical Significance:**

Sex and reproductive status influenced the RAAS of DP, with IF DP showing genotype‐independent higher ACE activity. These findings hold implications for sterilization practices in female dogs, and support sex and reproductive status as a source of variability in RAAS studies. Additionally, the frequency of the ACE gene variant was very high in this group of DP.

**Abbreviations**
AA2: aldosterone to angiotensin II ratio
ACE: angiotensin‐converting enzyme
ACE2: angiotensin‐converting enzyme 2
ALT‐S: alternative RAAS activation marker
DCM: dilated cardiomyopathy
DP: Doberman Pinschers
IF: intact female
IM: intact male
PRA‐S: plasma renin activity marker
RAAS: renin‐angiotensin‐aldosterone system
SF: spayed female

## 1 INTRODUCTION

The renin‐angiotensin‐aldosterone system (RAAS) is a complex neurohormonal cascade that regulates blood pressure, fluid balance, electrolyte balance, and inflammation.^1^, ^2^ The terminal metabolite of the cascade, angiotensin II, mediates classical pathway effects of vasoconstriction, sodium retention, aldosterone synthesis, and inflammation.^1^ An alternative RAAS pathway counterbalances these actions of angiotensin II with vasodilatation, natriuresis, and anti‐inflammatory effects through enhanced formation of angiotensin 1‐7 from angiotensin I and II.^1^

Estradiol is emerging as an important modulator of the RAAS in women, and the hormonal changes that occur during menopause appear to explain the increase in cardiovascular disease after the sixth decade.^3^, ^4^, ^5^, ^6^ Current research suggests that estradiol modulates various components of the RAAS to enhance angiotensin‐mediated inflammation during infection or trauma, which may explain the better ability of women of child‐bearing age to fight infections.^3^ Once the stimulus is resolved, however, estradiol then acts as a switch to promote vasodilatation, natriuresis, and anti‐inflammatory effects through the alternative RAAS pathway and activation of the angiotensin II receptor type 2.^3^, ^5^ Cascade changes resulting from estradiol influence in women are complex and include increases in angiotensinogen, angiotensin II receptor type 2 density, natriuretic peptides, and angiotensin 1‐7, accompanied by decreases in renin concentration, angiotensin‐converting enzyme (ACE) activity, angiotensin II receptor type I density, and aldosterone production.^6^ Removal of the cardioprotective effect of estradiol with onset of menopause dysregulates the RAAS, resulting in unopposed classical pathway effects of vasoconstriction, sodium retention, and inflammation, such that the incidence of cardiovascular disease in postmenopausal women increases to results similar to those of men.^5^, ^6^

Sex differences in the RAAS cascade are largely unexplored in client‐owned dogs aside from a study that showed an effect of breed, sex, and age on the urine aldosterone: creatinine ratio.^7^ In that study, intact female (IF) dogs had significantly higher urine aldosterone: creatinine ratios than did spayed female (SF) dogs, intact male (IM) dogs, and neutered male dogs, but other aspects of the RAAS cascade were not evaluated.^7^ The RAAS is increasingly recognized as a complex system that can be influenced by a variety of factors, including breed, sex, age, and genetic polymorphisms, many of which have not been explored.^6^, ^8^, ^9^, ^10^, ^11^ Understanding these confounding variables may help explain contradictory findings of previous studies of ACE inhibitors in dogs with heart disease, and is critical to the design of future studies.^7^, ^12^, ^13^, ^14^ Additionally, knowledge about the impact of surgically‐induced estrogen deficiency on the RAAS could be relevant in veterinary medicine.

Our purpose was to explore sex and female reproductive status as influential variables on the RAAS by evaluating circulating RAAS metabolites and enzyme activities in healthy, mature, IF, SF, and IM Doberman Pinscher (DP) dogs. We sought to comprehensively evaluate the circulating RAAS in these dogs using equilibrium dialysis to provide a global assessment of the cascade and build on previous work reporting urine aldosterone concentrations by ELISA methodology.^7^ We hypothesized that the RAAS profile of IF DP would differ from that of SF and IM DP.

## 2 METHODS

A convenience sample of 18 clinically healthy, mature DP (6 IF, 6 SF, 6 IM) was used. Residual serum from samples that were collected as part of an observational study evaluating the cardiac function of dogs eating grain‐free and grain‐inclusive diets^15^ was used to evaluate core RAAS metabolites (angiotensin I, angiotensin II, angiotensin III, angiotensin IV, angiotensin 1‐7, angiotensin 1‐5, aldosterone) and the enzyme activities of ACE and ACE2. Dogs were genotyped for variants associated with dilated cardiomyopathy (DCM) in this breed, including DCM1 (pyruvate kinase dehydrogenase 4) and DCM2 (titin),^16^ as well as for the known ACE gene variant^17^, ^18^ (https://cvm.ncsu.edu/nc-state-vet-hospital/small-animal/genetics/submit-dna-testing/). Sample collection was approved by the University of Florida IACUC committee (#201810504), and client consent was obtained from owners.

The inclusion of a single breed (DP) was intended to minimize variability that could be introduced by including dogs of multiple breeds. Dogs were included in the study if they were eating a grain‐inclusive diet, were between 18 and 96 months of age, and were apparently healthy based on history, physical examination, 20‐minute ECG monitoring during echocardiography, and echocardiography results using breed‐specific reference values.^19^ The age range was chosen to avoid sexually immature and geriatric dogs. Medications other than heartworm prevention were not allowed. Intact female DP were not in estrus, not pregnant, and not lactating at the time of sampling. Dogs were fasted before blood collection between 10 am and 2 pm and serum was removed immediately after centrifugation. Samples were stored at −80°C until batch analysis.

### 2.1 Equilibrium dialysis to assess the RAAS

The equilibrium concentrations of 6 different angiotensin peptide metabolites (angiotensin I, angiotensin II, angiotensin III, angiotensin IV, angiotensin 1‐5, angiotensin 1‐7) and aldosterone were quantified by liquid chromatography‐mass spectrometry/mass‐spectroscopy (LC‐MS/MS), performed by a service provider laboratory (Attoquant Diagnostics, Vienna, Austria), using previously validated and described methods.^14^ Analyte concentrations were reported in pmol/L and the lower limit of quantification was reported for each metabolite. The activities of ACE and ACE2 were measured using a kinetics approach with spiked substrate.^20^, ^21^ Activity of ACE was determined by measuring the formation of angiotensin II after addition of angiotensin I in the presence and absence of an ACE inhibitor and a chymase inhibitor to determine the ACE‐inhibitor sensitive fraction of angiotensin II generation. Activity of ACE2 was determined by measuring the formation of angiotensin 1‐7 in the presence and absence of an ACE2 inhibitor using recombinant human ACE2 as a reference standard. The ratio of angiotensin II to angiotensin I was calculated as a marker for ACE activity (ACE‐S).^14^ Angiotensin I and angiotensin II were summed as a marker for plasma renin activity (PRA‐S).^22^ The ratio of aldosterone to angiotensin II (AA2) was calculated as a unitless indicator of adrenal responsiveness to angiotensin II stimulation of aldosterone release.^14^ The sum of angiotensins 1‐7 and 1‐5 divided by the sum of angiotensins I, II, 1‐7, and 1‐5 was calculated as a unitless marker of renin‐independent alternative RAAS activation (ALT‐S). The angiotensin 1‐7/angiotensin I ratio was calculated to account for overall RAAS activity and aid interpretation of the balance of angiotensin 1‐7 formation and breakdown. This approach was necessary considering the high prevalence of the ACE gene variant in this group of dogs and the impact of overall RAAS activation on all downstream metabolites that result from metabolism of angiotensin I.

### 2.2 Statistical analysis

Demographic data including genotype, weight, and age were reported for each sex group as median (minimum, maximum), except for genetic status which was reported as observed frequencies (n; %). A priori power analysis was not performed because the study was exploratory. Proportions of genetically positive (homozygous or heterozygous, all variants) or negative (wildtype) dogs were compared among sex groups using a chi‐square test. The distributions of age (months), body weight (kg), and RAAS metabolites, ratios, and enzymes were compared among sex groups using the nonparametric Kruskal‐Wallis test followed by Dunn's test for multiple comparisons. Multiple linear regression was used to adjust the analysis for potential confounding effects of age on investigated associations among sex groups and RAAS variables (rank data). Using linear regression, dogs were assigned into 1 of 2 age groups based on the median distribution (i.e., 19‐38 months, n = 10; 46‐96 months, n = 8). Metabolite concentrations below the lower limit of quantification were entered as half of the lower limit of quantification for statistical comparisons. *P* < .05 was considered significant.

## 3 RESULTS

Most dogs (17/18) were ACE variant positive, whereas fewer dogs were positive for DCM1 (6/18) or DCM2 (7/18; Tables 1 and 2). Age was higher in IF DP and SF DP compared to IM DP but the difference among groups did not reach statistical significance (Table 2; *P* = .09). Weight and the proportions of DCM1, DCM2, and ACE variant positive and negative DP were not significantly different among sex groups (*P* > .2).

**TABLE 1:** Number of dogs with each genotype for each tested variant by sex

Genotype	Variant	IF	SF	IM
Wildtype	DCM1	3	4	5
	DCM2	6	2	3
	ACE	0	1	0
Heterozygous positive	DCM1	3	2	1
	DCM2	0	3	1
	ACE	3	2	2
Homozygous positive	DCM1	0	0	0
	DCM2	0	1	2
	ACE	3	3	4

Abbreviations: IF, intact female; IM, intact male; SF, spayed female.

**TABLE 2:** Comparison of genetic status, age, weight, RAAS metabolite concentrations, metabolite ratios, and enzyme activities of ACE and ACE2 among sex groups from univariate analysis

Variable	IF n = 6	SF n = 6	IM n = 6	*P*
DCM1 and DCM2 variant negative	3 (50)	1 (17)	3 (50)	.39
DCM1 or DCM2 variant positive	3 (50)	5 (83)	3 (50)	
ACE gene variant positive	6 (100)	5 (83)	6 (100)	.35
ACE gene variant negative	0 (0)	1 (17)	0 (0)	
Age (mo)	43.5 (22.0, 54.0)	49.0 (31.0, 96.0)	24.0 (19.0, 57.0)	.09
Body weight (kg)	34.4 (24.6, 40.8)	29.5 (23.7, 37.9)	37.0 (32.9, 39.3)	.2
ACE activity (ng/mL)	656 (437, 784)	411 (287, 451)*	365 (276, 1200)*	.02
ACE‐2 activity (ng/mL)	66.5 (31.2, 97.8)	60.6 (28.5, 73.4)	55.7 (31.7, 109.2)	.95
Angiotensin I (pmol/L)	158.7 (51.5, 248.1)	63.1 (18.5, 148.5)	88.5 (34.4, 220.2)	.23
Angiotensin II (pmol/L)	40.1 (18.9, 117.2)	20.9 (6.3, 99.9)	37.3 (7.6, 88.7)	.25
Angiotensin III (pmol/L)	9.3 (2.8, 41.7)	6.4 (3.3, 27.9)	12.2 (3.3, 38.0)	.67
Angiotensin IV (pmol/L)	7.3 (3.5, 20.7)	4.6 (2.1, 19.8)	9.1 (2.1, 26.6)	.56
Angiotensin 1‐5 (pmol/L)	74.0 (32.2, 221,6)	37.6 (10.2, 107.7)	68.4 (26.5, 107.2)	.58
Angiotensin 1‐7 (pmol/L)	31.3 (21.9, 55.2)	22.0 (10.1, 39.3)	26.7 (9.5, 55.7)	.51
Aldosterone (pmol/L)	78.7 (27.9, 224.4)	54.5 (18.1, 288.4)	121.3 (5.0, 173.1)	.67
PRA‐S (pmol)	207.6 (85.9, 331.3)	83.0 (24.8, 248.4)	125.8 (42.0, 309.0)	.17
AA2	1.6 (0.6, 4.8)	2.6 (1.4, 3.9)	2.4 (0.6, 8.9)	.88
Angiotensin 1‐7/angiotensin I	0.24 (0.16, 0.47)	0.34 (0.26, 0.55)	0.27 (0.25, 0.38)	.06
ACE‐S	0.3 (0.1, 0.6)	0.3 (0.3, 0.6)	0.4 (0.2, 0.5)	.89
ALT‐S	0.6 (0.3, 0.8)	0.8 (0.5, 0.9)	0.7 (0.5, 0.8)	.19

*Note*: The data are reported as median (minimum, maximum) except for genetic status which is reported as number of dogs (%). *P* values are unadjusted from Kruskal‐Wallis test and * indicates groups that are significantly different from IF after Dunn's test. Results of multivariable analysis can be found in the text of the results section.

Abbreviations: IF, intact female; IM, intact male; SF, spayed female.

In the univariable analysis (Table 2), ACE activity was different among sex groups, and it was highest in IF DP (*P* = .02). The angiotensin 1‐7/angiotensin I ratio was lower in IF DP compared to SF DP or IM DP, but the difference among groups did not reach statistical significance (*P* = .06). Angiotensin I, PRA‐S, and angiotensin II activity were higher in IF DP compared to SF DP, but the differences among groups were not significant (*P* ≤ .25). Finally, serum aldosterone concentrations were not different among sex groups (*P* = .67).

Using multivariable linear regression analysis, ACE activity was highest in IF DP compared to SF DP (*P* = .01) or IM DP (*P* = .04), after controlling for age. In addition, after controlling for age, the angiotensin 1‐7/angiotensin I ratio was lower in IF DP compared to SF DP (*P* = .01), but no difference was found between IF DP and IM DP (*P* = .32). The remainder of the RAAS variables and ratios also were not different among sex groups before or after controlling for age.

## 4 DISCUSSION

Sex and female reproductive status affected some components of the RAAS cascade in this small group of DP. The largest group differences were higher ACE activity in IF DP compared to SF DP and IM DP after controlling for age. These group differences were independent of the ACE variant, for which all but 1 dog was positive. Previous studies showed that dogs with the ACE gene variant have lower directly measured ACE activity and higher magnitude of aldosterone breakthrough after ACE inhibitor treatment compared to dogs without the variant.^14^, ^17^ High ACE activity in IF DP contrasts with reports of low ACE activity in premenopausal women, where estradiol appears to downregulate ACE.^6^, ^23^ Our results might not be directly comparable to studies in women because of possible confounding and unexplored effects that phase of reproductive cycle could introduce in both species, as well as potential differences between menopause and surgically‐induced reproductive hormone deficiency. Additionally, no studies in women have used the equilibrium dialysis approach for circulating RAAS analysis, and thus the use of different methodologies limits study comparisons.

A previous study reported higher urinary aldosterone concentrations in IF dogs compared to other sex groups,^7^ but we did not find significantly higher serum aldosterone concentrations in IF DP compared to the other sex groups after controlling for age. Serum aldosterone concentrations are not always correlated with urine aldosterone concentrations, however, and other factors such as competition by progesterone for the mineralocorticoid receptor could affect the relationship between serum and urine aldosterone concentrations.^6^ Alternatively, our study may have been underpowered to detect differences among groups. Although we did not find sex group differences for aldosterone, we did find numerically (but not statistically) higher concentrations of other metabolites that are associated with classical RAAS activation in IF dogs compared to SF dogs (e.g., angiotensin I and II). Interestingly, the aldosterone concentrations for all sex groups in our study were notably higher than aldosterone concentrations reported for healthy purpose‐bred dogs using the same methodology.^24^ An influence of ACE variant positivity on aldosterone concentrations could have occurred in our study population, because nearly all DP had this variant, which is associated with a higher magnitude of aldosterone breakthrough after ACE inhibition in dogs with heart disease.^14^ Other reasons for the differences among studies could be related to sampling, environment, or breed.

Although the frequency of the ACE variant in our dogs was very high, nearly all of the dogs were genetically positive and so this factor likely did not impact the sex group differences for ACE activity. Despite different ACE activities, the surrogate marker for this enzyme (ACE‐S), which is the ratio of angiotensin II to angiotensin I, was not different among groups. This seemingly discordant finding supports non‐ACE generation of angiotensin II in the circulation,^14^, ^25^ although it was not directly measured in our study. Non‐ACE enzymes, such as chymases, are capable of producing angiotensin II to maintain stable concentrations when ACE activity is low, thus explaining unaltered ACE‐S, which represents the end result of angiotensin II formation relative to angiotensin I without consideration of the various pathways that produce it. The method we used to directly measure ACE activity is a better assessment of ACE activity than the surrogate ratio ACE‐S because it accounts for chymase‐generated angiotensin II by using both a chymase inhibitor and an ACE inhibitor to determine only the ACE inhibitor‐sensitive fraction.

A previous study showed predispositions for the ACE variant in several breeds known to have genetically‐based heart disease (e.g., Irish Wolfhounds, Cavalier King Charles Spaniels), but there were insufficient numbers of DP in that study to assess breed predisposition.^18^ Our study suggests a high prevalence of the ACE variant in DP. The importance of this variant to genetically‐based DCM in the DP requires study.

The lower angiotensin 1‐7/angiotensin I ratio in IF DP compared to SF DP after controlling for age, indicates that there was relative classical RAAS predominance in IF DP and relative alternative RAAS predominance in SF DP. Relating angiotensin 1‐7 to angiotensin I is important to account for overall RAAS influence, which can impact downstream metabolite formation globally. Interpretation of 1 metabolite without consideration for the others in the cascade could be misleading. The ALT‐S calculation, which is an estimate of the entire alternative RAAS arm, was also lower in IF DP, although the differences among sex groups were not significant. The finding that angiotensin 1‐7/angiotensin 1, but not ALT‐S, was significantly lower in IF DP compared to SF DP could have been a consequence of small sample size or impact of the ACE variant. The major formation enzyme for the alternative RAAS (ACE2), was not different between groups, indicating that the generation of angiotensin 1‐7 mediated by this enzyme was not different among groups. The ACE enzyme however also contributes to angiotensin 1‐7 concentrations because it catalyzes the breakdown of angiotensin 1‐7 to angiotensin 1‐5, in addition to its well‐known role of converting angiotensin I to angiotensin II. Therefore, lower ACE activity is expected to stabilize angiotensin 1‐7 concentrations by decreased degradation. The higher angiotensin 1‐7/angiotensin I ratio in conjunction with unchanged ACE2 in SF DP compared to IF DP therefore is consistent with decreased metabolism of angiotensin 1‐7 as a result of lower ACE activity in this sex group.

Three measures of the classical RAAS arm (angiotensin I, angiotensin II, and PRA‐S, which is a surrogate for renin) were higher in IF DP compared to SF DP, but statistical significance among sex groups was not observed. Because this result could be a consequence of chance, this finding requires study in a larger group of dogs, but is consistent with the other study findings suggesting that IF DP appear to have more classical RAAS influence whereas SF DP appear to have more alternative RAAS influence. Although this blunting might seem beneficial (and indeed is so in the setting of heart failure‐related chronic overactivation), the production of RAAS metabolites in a healthy animal is a desired response to intermittent cardiovascular stressors and infection. A key difference between health and disease might lie in the dysregulation or persistent activation seen in cardiac disease states compared to appropriate modulation and situation‐specific regulation in health.^1^, ^23^ The alternative RAAS arm typically is considered beneficial in the setting of the RAAS‐activated state of heart disease because it indicates higher concentrations of angiotensin 1‐7 (which promote vasodilatation, natriuresis, diuresis, and anti‐inflammatory effects) and lower concentrations of angiotensin II and other classical metabolites (which promote vasoconstriction, sodium and water retention, and inflammation). Our findings need to be confirmed in a larger study in which the RAAS is challenged, but they could be consistent with the conclusion that the classical RAAS is less primed to address stimuli in SF dogs, possibly as a result of estrogen deficiency, or they could indicate a beneficial state with predilection for the alternative RAAS pathway.

Our study had several limitations. This initial investigation into the effect of sex on the RAAS was designed as an exploratory study with small numbers, and so the resulting power to detect group differences was low for some metabolites. For example, although median values for angiotensin I and PRA‐S were higher in IF DP than SF DP, observed differences did not reach statistical significance because the study sample was small (power was 57% and 48%, respectively). If these differences were real, a larger sample size of 11 and 13 dogs per group, respectively, would be required to reach statistical significance. Consequently, significant and nearly significant findings might be worthy of additional study in a larger group of dogs. The activities of ACE reported in our study are specific to the equilibrium dialysis approach and are not directly comparable to studies utilizing other methodologies. Another limitation of our study is the use of a single breed, which restricts applicability to other breeds, especially in light of breed predispositions for, and the potential influence of, the ACE variant.^14^, ^18^ Neutered male dogs also were not evaluated in our study because studies in people have indicated that estradiol is the major influencer of the RAAS and testosterone appears to have little effect on the cascade. Enrolled dogs were apparently healthy based on history, physical examination, limited ECG monitoring, and echocardiography, but screening blood tests, thyroid testing, heartworm testing, Holter monitoring, blood pressure, and urinalysis were not performed. Another important consideration is that our study investigated the circulating RAAS, but evaluation of tissue RAAS might show different results. We proposed 1 interpretation of the data to be that SF DP might have less RAAS reserve (i.e., less ability to respond to stimuli with RAAS activation), but this possibility was not evaluated in our study. Lastly, although no IF dogs were pregnant, lactating, or in estrus, temporal changes related to phase of the reproductive cycle were not evaluated in IF DP in our study and sex hormones, specifically estradiol, were not measured. Future studies should address hormonal alterations over time in IF dogs.

Our results indicate that sex and female reproductive status influence the RAAS of DP, with IF DP showing higher ACE activity than SF DP and IM DP. The potential role of estrogen on the RAAS and its relationship to lower ACE activity will need to be investigated in future studies, but based on studies in people, estrogen could be a cause of the group differences.^2^, ^23^ These findings have implications for sterilization practices in female dogs and support sex and reproductive status as sources of variability in RAAS studies. Our study also found a high frequency of the ACE variant in this group of DP. In light of sex group differences in ACE activity, this finding suggests an interplay of breed‐related genetic variation with sex and reproductive status, and contributes to our understanding of modifying influences on the RAAS that might have impacted results of previous studies and should be considered in future study designs.

## CONFLICT OF INTEREST DECLARATION

Dr. Adin acknowledges research support from Nestle Purina PetCare and is a consultant and sponsored lecturer for Ceva Animal Health and Boehringer Ingelheim. Dr. Adin is a member of the Scientific Advisory Panel for the Translational RAAS Interest Group but recused herself during this funding cycle. Dr. Hernandez does not have a conflict of interest.

## OFF‐LABEL ANTIMICROBIAL DECLARATION

Authors declare no off‐label use of antimicrobials.

## INSTITUTIONAL ANIMAL CARE AND USE COMMITTEE (IACUC) OR OTHER APPROVAL DECLARATION

Approved by University of Florida IACUC, #201810504.

## HUMAN ETHICS APPROVAL DECLARATION

Authors declare human ethics approval was not needed for this study.
